# Pattern and Determinants of Gestational Weight Gain an Important Predictor of Infant Birth Weight in a Developing Country

**DOI:** 10.5539/gjhs.v6n4p148

**Published:** 2014-04-14

**Authors:** Olapeju Adefunke Esimai, Ebenezer Ojofeitimi

**Affiliations:** 1 Department of Community Health, Obafemi Awolowo University, lle-lfe, Nigeria; 2 Department of Community Health, Ladoke Akintola University, Osogbo, Nigeria

**Keywords:** gestational weight gain, infant bith weight, pepregnant body mass index

## Abstract

The study aimed to determine correlates of gestational weight gain and infant birth weight of pregnant women attending antenatal clinics in public primary health care facilities in lfe Central and East Local Government Areas of Osun State, Nigeria. Over 1000 women were recruited during booking and antenatal clinic and followed up till delivery. Chi square was used in the bivariate analysis of association between gestational weight gain, pre pregnancy BMI and demographic characteristics. The correlates of gestational weight gain and infant birth weight were determined by linear regression analysis.

Eight percent are underweight, 10.3% are overweight or obese, 78% had a weight gain less than 7kg and 0.5% had a weight gain above 11.5kg. Ninety seven percent gained less than recommended weight, only 3% of the women gained the recommended weight for their pre pregnant BMI mostly the obese women. Twenty eight percent of the women had infant weight within normal (2.5kg and above). The infant weight increases with the gestational age, maternal age and parity but decreases with gestational weight gain though not significant. Maternal age and parity were significant predictors of gestational weight gain and pre pregnancy BMI was a significant predictor of infant birth weight.

The gestational weight gain and infant weight reduces as the pre pregnant BMI increases. Most of the women had low birth weight babies. There is a need to educate mothers on good weight before conception in order to improve birth outcome in view of other factors not looked into in the present study.

## 1. Introduction

The inadequacy of nutritional status within reproductive age and pregnancy is an important health and nutritional problem among women and their children; this might bring undesirable consequences to reproductive health, as well as negatively contribute to child development, with reflections on birth conditions and morbid mortality rates (Carvalho Padilha et al., 2009). Pre-gestational maternal nutritional status and gestational weight gain have been studied systematically, due to the growing prevalence of deviations from their normal values and their determinant role in gestational outcomes (National Research Council/Institute of Medicine, 2007).

The increase in body mass index (BMI) among pregnant women worldwide has become one of the most significant public health concerns (Yazdani et al., 2012). The Institute of Medicine (IOM) provided target ranges of recommended weight gains by pre pregnancy body mass index (BMI; in kg/m^2^). The IOM divided BMI into 4 categories using IOM criteria: underweight (BMI <19.8), normal weight (BMI 19.8-26), overweight (BMI 26.1-29), and obese (BMI>29) (Institute of Medicine 1990). There are recommended ranges of total weight gain for pregnant women by pre-pregnancy BMI known as the “IOM’s recommended weight-gain ranges” The IOM recommends a gain of 12.5kg to 18.0 kg during pregnancy as optimal, a gain of 11.5-16.0kg in women with normal BMI and of 7.0-11.5kg for high BMI (overweight) and less than 7kg for obese women (Rasmussen & Yaktine, 2009). Women in less developed south-eastern Asia, including Viet Nam, tend to be smaller and to gain less weight during pregnancy on average than Caucasian women in Europe or the United States (Food and Agriculture Organization of the United Nations, 2010). The need to review whether the current anthropometric recommendations for pregnant women of the United States National Academy of Sciences Institute of Medicine (IOM), which are based on data from western countries are appropriate for preventing adverse pregnancy outcomes across populations everywhere, including south-east Asia was emphasised in a previous study (Ota et al., 2011). Maternal age, serum triglycerides and blood glucose, age at menarche, and adequacy of energy consumption have been reported to be associated with gestational weight gain. For each increase of one year in the woman’s age, there was an increase of 0.631kg in weight gain (Rodigues et al., 2008).

Low birth weight is a public health problem (Wise, 2010), and complicates around 17% of all births. It is the major risk factor for mortality in early infancy. Half of all low birth weight babies are born in South Central Asia, where more than a quarter (27%) of all infants weighs less than 2,500 g at birth. Low birth weight levels in sub-Saharan Africa are around 15 per cent. Central and South America have on the average much lower rates (10%), while in the Caribbean the level (14%) is almost as high as in sub-Saharan Africa. About 10% of births in Oceania are low birth weight births (Monawar, Hosain, Chatterjee, Begum, & Saha, 2006). Several factors such as mother’s genetic characteristics, social cultural demographic, behavioural factor, pre pregnancy body mass index, gestational weight gain etc contribute to birth weight (Carvalho Padilha et al., 2009). Neonatal birth weight is an important determinant of infant’s well being, and maternal BMI during pregnancy is one modifiable factor influencing neonatal birth weight outcome (Upadhyay, Bicche, Sherpe, Strestha, & Panta, 2011). Maternal risk factors for having an infant with LBW in general include young age, unmarried marital status, less education, lower income, smoking, poor nutrition, and having had a previous infant with LBW (kabir, Comolly, Clancy, Cohen & Kohn, 2009). It was suggested that environmental factors play a significant role too (Fahrija et al., 2010). Yilgwan, Abok, Yinnang and Vajime (2009) in a previous study had reported an association between multiparity and low birth weight. Besides, multiparity and higher number of births is a common feature seen in women of low socioeconomic status a factor associated with low birth weight (Carvalho Padilha et al., 2009). Previous study have shown that gestational weight gain and age of the mother at the time of delivery played an important in determining the birth weight of the infant delivered at Patan hospital (Shrestha, Sunawa, Bhanday, & Sharma, 2010). A study carried out in Vietnam showed that low maternal BMI and a weight gain of <10kg during pregnancy, especially in combination, put women at risk of having infants too small for gestational age (Ota et al., 2011)

Information on pattern of weight gain in pregnant women in Nigeria is scarce and studies that demonstrate relationships between weight gain, its correlates and infant birth weight in developing countries are few. The purpose of the study is to determine pattern of weight gain in pregnant mothers and examine maternal demographic and anthropometric variables that influence weight gain during pregnancy and infant birth weight in an urban setting. The study is carried out with a view to develop programmes to assist expectant mothers to achieve recommended weight gain during pre -pregnant stage and gestation in order to keep optimum weight to ensure well being of the baby.

## 2. Methodology

The observational study was initiated in year 2000. Pregnant women in all public primary health care facilities in urban communities in Ife Central and Ife East LGAs in Osun state, Nigeria were enrolled for the study. Between the years 2000 and 2001, a cohort of 1000 women was recruited to participate in the study. They enrolled during the booking clinic and routine antenatal clinic and followed up to delivery. Permission was obtained from the Director Primary Health Care at the 2 LGAs. Ethical approval was given by the ethical committee in the State Ministry of Health. Every participant had the right to participate except subjects with complicated pregnancy and free to leave the study whenever they want to. The women were informed about the study and their consent and that of their spouses were sort before participating in the study. They were assured confidentiality of the information given.

Estimated gestational age was calculated based on last menstrual period. Baseline weight and height were recorded during the first visit. Weight was measured using calibrated scale accurate to within 0.5kg while subject were wearing light clothing and height measured using calibrations on the wall in meters. Pre pregnancy weight was based on weight measured during the first 2 months of pregnancy. Information on maternal age, parity, education and occupational status were collected. Maternal pre pregnancy BMI were categorised based on 1990 IOM into <19.8 kg as underweight, 19.8-26kg (normal weight), 26-29.9kg (overweight) and ≥30 kg as obese. Total weight gain in pregnancy was estimated by subtracting the pre pregnancy weight from last measured weight before or at delivery. Weight gain less than 7kg was considered low and weight gain above 7kg as high weight gain for purpose of analysis. Outcome measures are gestational weight gain and infant birth weight. Chi square was performed as appropriate, p value less than 0.05 considered as being significant. Linear regression analysis was used to examine relationship between gestational weight gain, infant birth weight and predictor variables such as pre pregnancy BMI, maternal age, parity and gestational age (trimester), education and occupational status are adjusted.

## 3. Results

**Table 1:** Characteristics of study population

Variables	Mean ±SD
Maternal age (yrs.)	24.19± 0.29
Pre pregnancy maternal weight(kg)	56.84±0.29
Maternal height (m)	1.58±0.01
Pre pregnancy BMI(kg/m²)	22.89±0.12
**Maternal characteristics**	**n (%)**
Age group(n=590)	
18	46 (7.8)
19-35	533(90.3)
>35	11(1.9)
Parity (n=590)	
Nulliparous	187(32.3)
Multiparous	376(63.7)
Grandmultiparous	27(4.6)
Educational status (n=522)	
Lower	408(78.2)
Higher	114(21.8)
Occupational status(n=530)	
Unskilled	403(76.0)
Skilled	85(16.6)
Professional	39(7.4)
Gestational age(trimester)	
First	36(6.1)
Second	275(46.6)
Third	279(47.3)

Mean age was 24.2±6.29 with a range of 18-40 years. Mean height (1.58±0.003), mean pre pregnancy weight (56.8±0.29), mean pre pregnancy BMI 22.89±0.12. Majority (67.7%) were multiparous while 32.3% are nulliparous, (78.2%) had lower educational status while 21.8% had higher educational status, and majority (76.0%) are unskilled while 7.9% are professionals’ mainly civil servant. Most of the women (46.6% & 47.3%) were seen at the second and third trimester.

**Table 2:** Anthropometry data of the study population

BMI	Freq (%)
<19.8	47 (8.0)
19.8-26	482 (81.7)
26.1-29	46 (7.3)
>29	15 (2.5)
Gestational Weight gain	
<7.0	572 (97.0)
7-11.5	15(2.5)
≥11.5	3(0.5)
Birth weight (kg)	
<2.5	424(71.9)
>2.5	166(28.1)

Majority (81.7%) had normal BMI, 7.8% were overweight and 2.5% were obese. Ninety seven percent had low weight gain (<7kg), 3.0% had high weight gain (7-11.5kg and above). Majority (71.9%) had low birth weight babies (<2.5kg)

**Table 3:** Recommended gestational weight gain stratified by Body Mass Index

BMI/Wt gain	IOM guideline(kg)	Less than recommended	Recommended	More than recommended
Underweight	12.5-18	46(97.9)	1(2.1)	0(0.0)
Normal weight	11.5-16	479(99.6)	2(0.2)	1(0.2)
Overweight	7-11.5	45(97.8)	1(2.2)	0(0.0)
Obese	<7	0(0.0)	14(93.3)	1(6.7)
Total		570(96.6)	18(3.1)	2(0.3)

Pre pregnancy Body Mass Index was stratified by recommended weight gain. Majority (96.6%) gained less than recommended weight for their pre pregnancy BMI, 3.1% gained the IOM recommended weight gain and 0.3 % gained more than recommended weight. Two percent of the underweight women, 0.2% of normal weight and 2.2% of the overweight women gained recommended weight gain. Majority (93.3%) of the obese gained the recommended weight, 6.7% gained more than recommended weight.

**Table 4:** Association between maternal characteristics, nutritional status and weight gain in pregnancy

Maternal Characteristics/Nutritional status	Weight gain		Test statistics x²/fisher exact	P value

	Low	High		
*Age (years)*			10.31	0.001
18	41(89.1)	5(10.9)		
>18	531(97.6)	13(2.4)		
*Parous*			4.88	0.03
Nulliparous	177(94.7)	10(5.4)		
Multiparous	395(98.0)	8(2.0)		
*Educational status*				0.001
Lower	400(98.3)	7(1.7)	11.57	
Higher	104(92.0)	9(8.0)		
*Occupational status*			4.37	0.037
Unemployed	395(98.0)	8(2.0)		
Employed	120(94.5)	7(5.5)		
*Body Mass Index*				0.78
Low BMI	46(97.9)	1(2.1)		
Normal BMI	467(96.9)	15(3.1)		
High BMI	59(98.3)	1(6.7)		
*Gestational age*				0.30
First trimester	35(97.2)	1(2.8)		
Second trimester	264(96.0)	11(4.0)		
Third trimester	273(97.9)	5(2.1)		

The proportion of adolescent pregnant mothers that gained high weight was significantly higher than young and older women. Similarly the proportion of pregnant women with high level of education that gained high weight was more compared with women in low educational status. The proportion of employed pregnant women that gained high weight was more compared with unemployed women. However the proportion of women who were underweight, normal weight or overweight with high weight gain were similar in proportion.

**Table 5:** Linear regression result of predictor variables of gestational weight gain

Variables	β	P value
Maternal age (years)	1.39	0.005
Parity	-0.81	0.05
Gestational age	0.32	0.28
Pre -gestational BMI	-0.35	0.44

Maternal age and Parity are significant predictors of gestational weight gain (^β^coefficient=1.39, p =0.005; ^β^coefficient=-0.81, p =0.05.

**Table 6:** Linear regression results of predictor variables of infant birth weight

Variables	^β^coefficient	P value
Maternal age (years)	-0.09	0.45
Parity	0.09	0.47
Gestational weight gain	0.31	0.16
Pre gestational BMI	0.25	0.02
Gestational Age	0.45	0.61

Pre gestational BMI is a significant predictor of infant birth weight (^β^coefficient =0.25, p=0.02).

## 4. Discussion

The mean pre pregnancy weight gain in the study was 56.8kg which is much lower than the pre pregnancy weight of 63.4kg in a study conducted in Department of Paediatrics, and Obstetrics unit of University of Maiduguri Teaching Hospital (UMTH) (Baba et al., 2012). The difference could be due to the fact that the hospital is the largest health facility in the area, serves as a referral centre for the six states in north –east region and neighbouring countries of Chad, Cameroon and Niger Republics. The mean maternal height 1.58meters is comparable with mean maternal height of 1.63 meters in the UMTH study (Baba et al., 2012) as both populations may be of similar characteristics.

The mean pre pregnancy BMI is 22.9±0.12 was a little lower than 23.2kg and 23.7kg/m² reported in public maternity hospital in Rio de Janeiro and the UMTH study respectively (Carvalho Padilha et al., 2009; Baba et al., 2012). This could be as result of difference in study locations. The findings of more than three quarters of subjects having a normal pre pregnancy BMI (19.8-26 kg/m²) is an improvement on the report of 65.4% women had normal pre pregnancy BMI in a study conducted among women in vietnam (Ota et al., 2011). Few (8.0%) are underweight, 7.3% overweight and 2.5% obesity, this is far below the report of 26.1% of low BMI recorded in a previous study amongst women in Vietnam, but slightly higher than 8.5% high BMI. The difference may be due to the classification of low BMI as <18.5 and high BMI >25 in Asian countries where women tend to be smaller.

Most of the subjects with normal BMI gained less than the recommended weight gain for their pre pregnancy BMI, Only few (3.1%) gain recommended weight for their pre pregnancy BMI. Most of the women with low BMI (underweight) in the present study gained less than recommended weight (12.5-18kg), this is supported by the report of 78% of the pregnant women in Vietnam with low BMI gained more than 10 kg. This is in contrary to the report by Yadzani et al. (2012) of an inverse relationship between maternal BMI and weight gain during pregnancy. This could be due to a dissimilarity in the population characteristics and number of factors such as dietary, genetic and environmental factors. Most of the obese subjects gained the recommended weight and 6.7% gain more than 11.5kg. This observation is similar to the report of obese women gained less weight during pregnancy than normal or overweight women; yet about one-fourth of obese women still gained 35 pounds or more (Chu, Callaghan, Bish, & Angelo, 2009).

Slightly above a quarter of the women had neonates within acceptable weight (≥2.5kg) in the present study, this far lower than 86.5% reported in UMTH study (Ahmadu et al., 2012). This difference might be due to the sample size and mother-neonate pair used in the methodology. The proportion of mothers that had weight gain below the total weight gain during pregnancy that had low birth weight babies was high but not significant compared to proportion of mothers had total weight gain within normal or above. However a previous study conducted amongst pregnant females who registered in Baptist Hospital Eku, Delta state, Nigeria (2012) showed that weight gain in pregnancy has a positive linear relationship (correlation) with the birth weight. The dissimilarity may be due to the use of retrospective survey method and correlation method of analysis in the previous study.

The linear regression analysis showed that the predictor for gestation weight gain are maternal age and parity, this supports the report of a multivariable regression model of maternal pre pregnancy obesity as the strongest predictor of low GWG (obesity correlated with lowest GWG), followed by higher parity, African American or Hispanic racial identity, and higher maternal age (Chu et al., 2009).

Pre gestational BMI, a reflection of gestational weight gain can act as biomarker for infant birth weight. There was no significant relationship between gestational weight gain and birth weight in the present study, this was contrary to the finding in previous studies of the statistical significant relationship between weight gain in pregnancy and birth weight of baby (p<0.05) (Nwangwa, 2012). The difference might be in the use of ANOVA in previous studies and linear regression analysis in the present study. There was a significant association between pre pregnancy BMI and infant birth weight in the present study, this was contrary to the report of the UMTH study of maternal pregnancy BMI not significantly contributing to birth weight (Baba et al., 2012). However the finding of the present study was in agreement with the findings of study amongst Brazilian women attending public maternity hospital in Rio de Janeiro where the predictor variables of birth weight were total gestational weight gain, pre-gestational BMI, maternal age and number of prenatal care appointments (Carvalho Padilha et al., 2009). The finding of the present study also corroborate the report of maternal pregnancy BMI having strong association with birth weight of neonates in Asia (Upadhyyay, Bicche, Sherpe, Strestha, & Panta, 2011). The similarities may due to similar characteristics in the study population.

## 5. Conclusion

The study showed high gestational weight gain amongst young women and low gestational weight gain amongst the obese women. Maternal BMI contribute significantly to birth weight while gestational weight gain had no relationship with infant birth weight. The current intervention programme needs to be reviewed. However with caution so that young mothers don’t gain excessive weight because of possibility of obesity in near future. Factors like dietary intake, physical activity and behavioural factors need to be assessed as these may help in improving the effectiveness of the intervention programme in the future.

## Limitations

The study is observational hence drawing inferences with regard causal relationship was done with caution. Secondly, there was no assessment on dietary/food intake, physical activity and other behavioural factors. The study could not report on other outcome variables like status of children at birth and their anthropometric data because the data on these variables are not adequate. Almost half of the respondents could not be traced close to delivery or at delivery because many had to relocate because of communal crisis and some facilities could not be assessed during this communal crisis.
